# Supplementary material for: STEPS 4.0: Fast and memory-efficient molecular simulations of neurons at the nanoscale
Source: Front Neuroinform. 2022 Oct 26;16:883742. doi: 10.3389/fninf.2022.883742 (PMC9645802; doi:10.3389/fninf.2022.883742)
Supplement: Supplementary file 1 [file Presentation_1.PDF]

frontiers

Supplementary Material

1 METHODS

1.1 Mesh generation, partition and annotation

Due to the distributed nature of STEPS 4, a new mesh importing, partitioning and annotation solution has been added, supported by the Omega_h and Gmsh (Geuzaine and Remacle, 2009) libraries. Currently it supports importing Gmsh .msh meshes, with or without pre-partitioning. If the mesh is not pre-partitioned, the Omega_h backend in STEPS 4 performs a recursive bisection partitioning on the mesh at MPI rank 0, then distributes each mesh partition to the owner rank. If the mesh is pre-partitioned in Gmsh, each rank imports its associated partition directly from the file and then establishes the connectivity to other processes. In both cases, the number of MPI processes used in a STEPS 4 simulation should always be a power of 2 due to the restriction imposed by Omega_h. It is also worth mentioning that the two solutions apply different partitioning schemes, which may affect simulation performance drastically depending on the mesh morphology.

Another significant change on mesh preparation is the annotation of physical entities of the mesh such as "compartments" representing volumetric regions like the cytosol and extracellular space, and "patches" representing surface regions such as the cell membrane and intracellular membranes. In STEPS 3, the user provides a list of tetrahedron/triangle indices to create a compartment/patch of the mesh. The index lists are either generated on the fly during the simulation or generated in advance, stored externally then provided to the simulation. The user is expected to take full responsibility for the generation and maintenance of these lists. In STEPS 4, the annotation of compartment/patch is embedded into the mesh file itself by utilizing the physical tag functionality in Gmsh. As a general procedure, the user can create physical entities on the mesh using the Gmsh application and provide a string as the physical tag for each of the entities. These string tags can then be used as identifiers to create the compartments and patches in STEPS 4. If the anticipated boundaries of the compartments/patches are irregular, but can be described by a combination of enclosed polyhedral surfaces, then the user can also utilize the gmsh backend implementation of the PolyhedronROI[1] utility to embed the physical tags into associated mesh elements.

2 VALIDATIONS

2.1 Rallpack 1: Mesh refinement

We further examine the model convergence through mesh refining. Usually such studies start from a very coarse mesh and refine the solution through mesh splitting until results converge and residual errors plateau at numerical precision. However, the cable geometry is extreme: its length is 3-4 orders of magnitude bigger than its radius. This means that sensible mesh refinements split it only lengthwise. Since $V_{z_{min}}$ is the voltage trace where the current is injected and is dominated by the mesh size in the radial direction, it can be discarded in the current analysis. The focus is on $V_{z_{max}}$. Figure S1 presents the mse (units mV^2) in the typical log-log scale. As expected accuracy plateaus as it approaches numerical precision.

[1] https://github.com/CNS-OIST/STEPS_PolyhedronROI

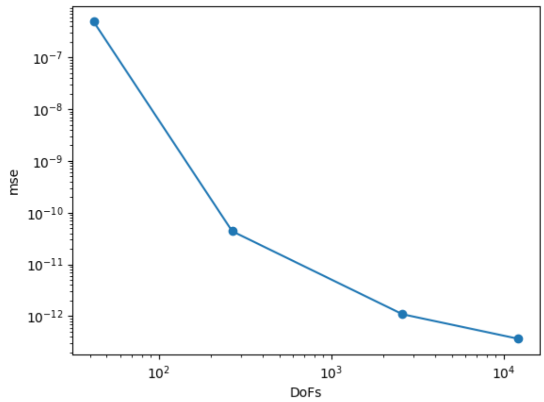

Figure S1. Convergence of the STEPS 4 trace $V_{z_{max}}$ to the analytical solution through mesh refinement in a log-log graph.

2.1.1 Rallpack 3: Distributions, means and std. deviations

Here we present the distribution plots for peak heights and time stamps. This is not meant to be proof that the two simulators produce the same results. It is to introduce the reader to the results with a general, qualitative overview.

Figure S3 illustrates average and standard deviation of the difference between STEPS (3 or 4) and the NEURON reference for peak heights and time stamps at $V_{z_{min}}$ and $V_{z_{max}}$. Peak heights match closely to the NEURON reference while peak time stamps slightly drift. Notice that this frequency difference is very small and we magnified it here by studying the difference between STEPS and NEURON for the sake of exposition. For example, peak number 17 occurs at around 250 ms and the drift is only ~0.6 ms. This small discrepancy is of no concern since even NEURON results slightly change by similar amounts depending on the time step of integration. Another interesting fact is that the first peak presents much smaller standard deviation compared to the others. This is due to the fact that all the simulations begin from the same starting point.

2.2 P-value distributions and discretization effects

Figures S4 to S7 illustrate all the distributions of the p-values obtained with the CVM test for all the peaks. They are grouped by trace position (z_{min}, z_{max}) and height and time stamp. The study considers 20000 simulations: 10000 for STEPS 3 and 10000 for STEPS 4. They are batched in 100 groups of 100 runs. Since batches are independent from each other each batch from STEPS 3 is tested (with the CVM test) versus each batch from STEPS 4 and a p-value is generated. In this way we produce 10000 independent p-values per peak. Since in Figures S4 to S7 the distributions appear uniform, we cannot refute the hypothesis that the samples are taken from the same population.

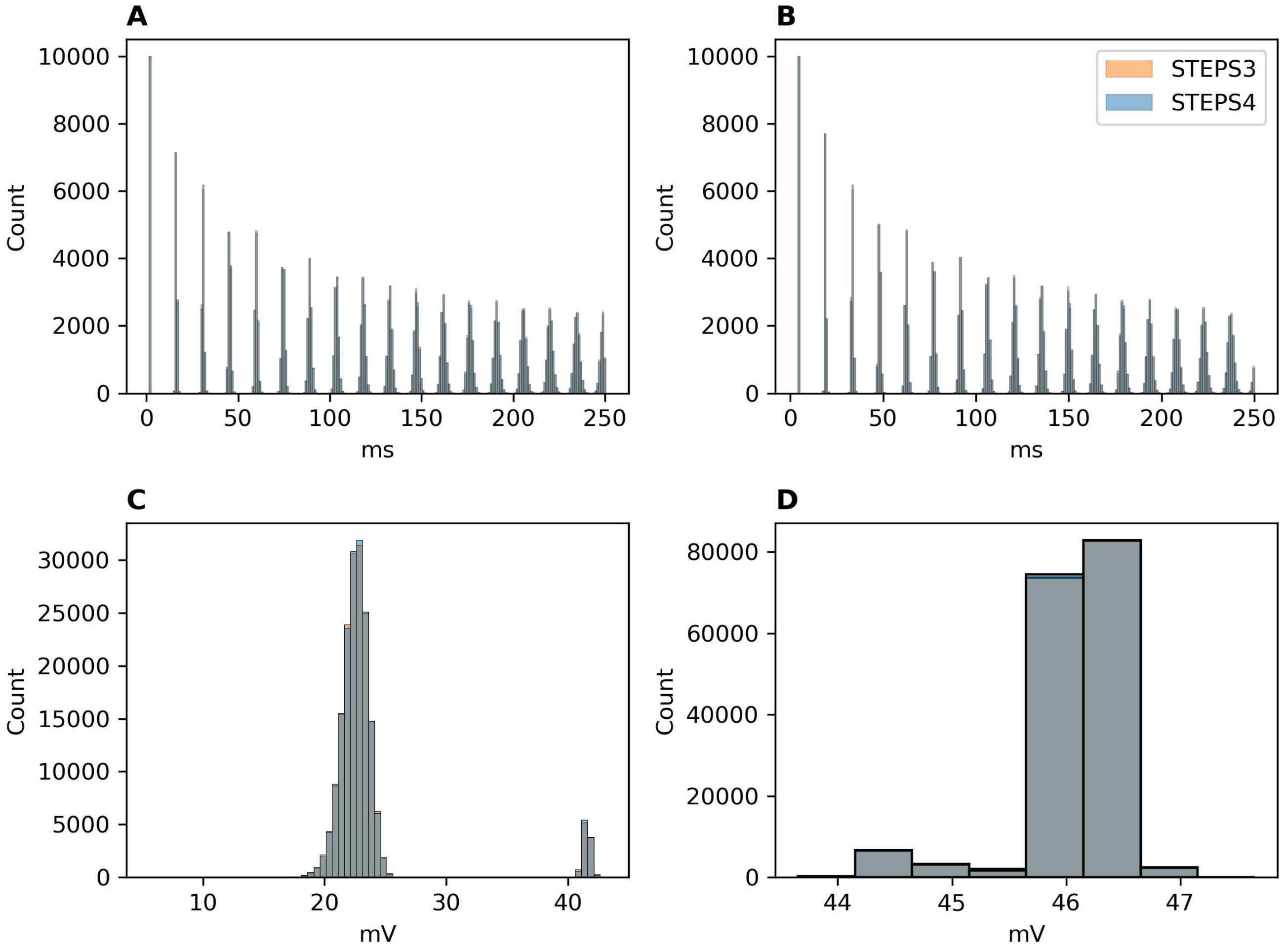

Figure S2. Distribution of peak time stamps (**A** and **B**) and heights (**C** and **D**) for STEPS 3 and STEPS 4 at z_{min} (**A** and **C**) and z_{max} (**B** and **D**). The plot for z_{min} presents two different peaks because the first spike in the spike train is consistently higher. In addition we notice that there is much less variance at z_{max} than z_{min}; perhaps due the large number of channels involved in active spike propagation compared to the smaller number of channels involved in spike generation. Qualitatively, STEPS 3 and STEPS 4 are in agreement.

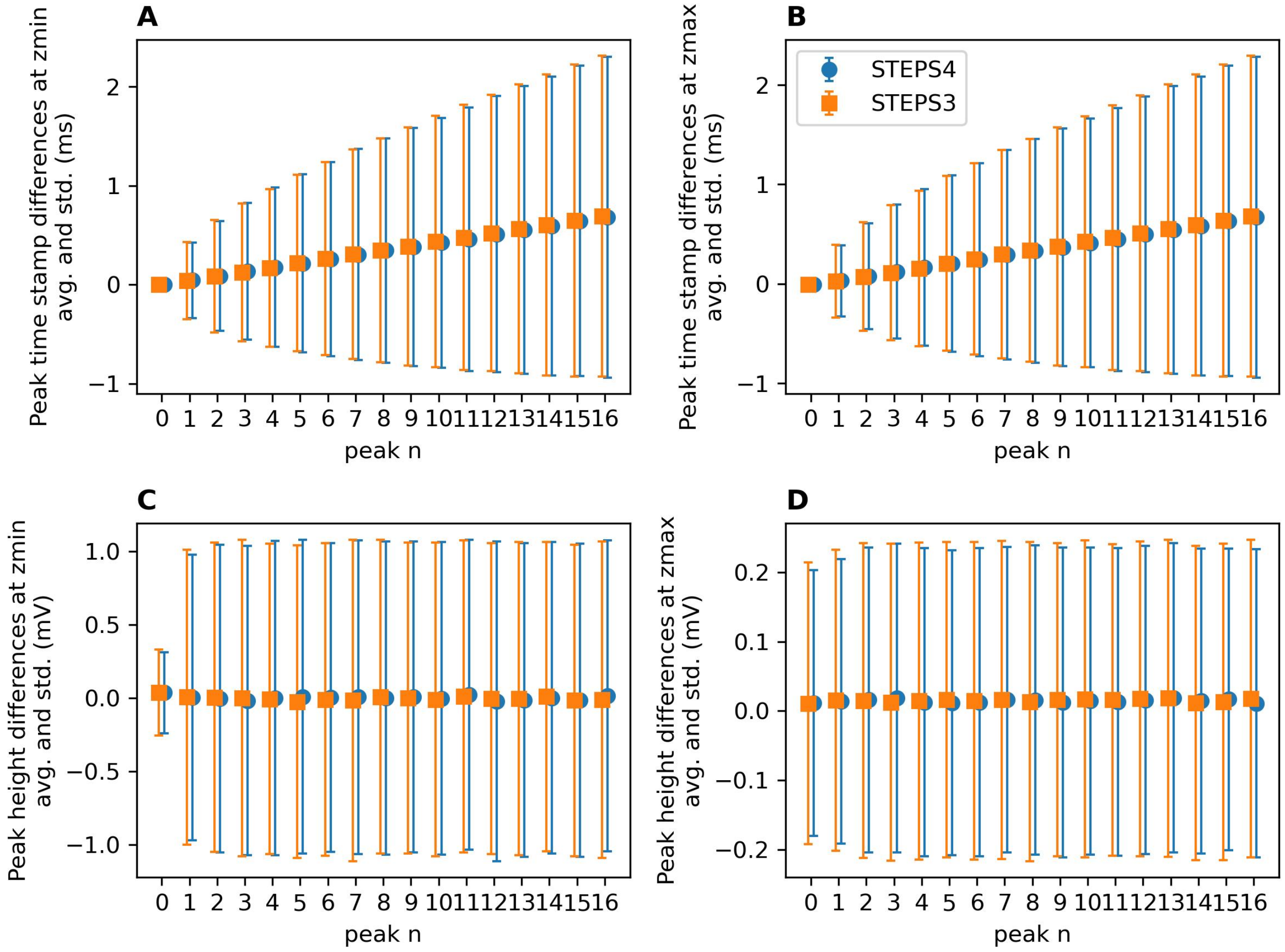

Figure S3. Mean and std. deviation of the difference between the peak time stamps (**A** and **B**) and heights (**C** and **D**) for STEPS (3 or 4) and NEURON reference at z_{min} (**A** and **C**) and z_{max} (**B** and **D**). The first peak is deterministic: appears always at the same time and the std. deviation is small. After, randomness enters the system and peaks tend to go out of phase, lowering peaks in the distribution analysis and widening the bases. Means and std. deviations climb as time passes. STEPS 3 and STEPS 4 results overlap.

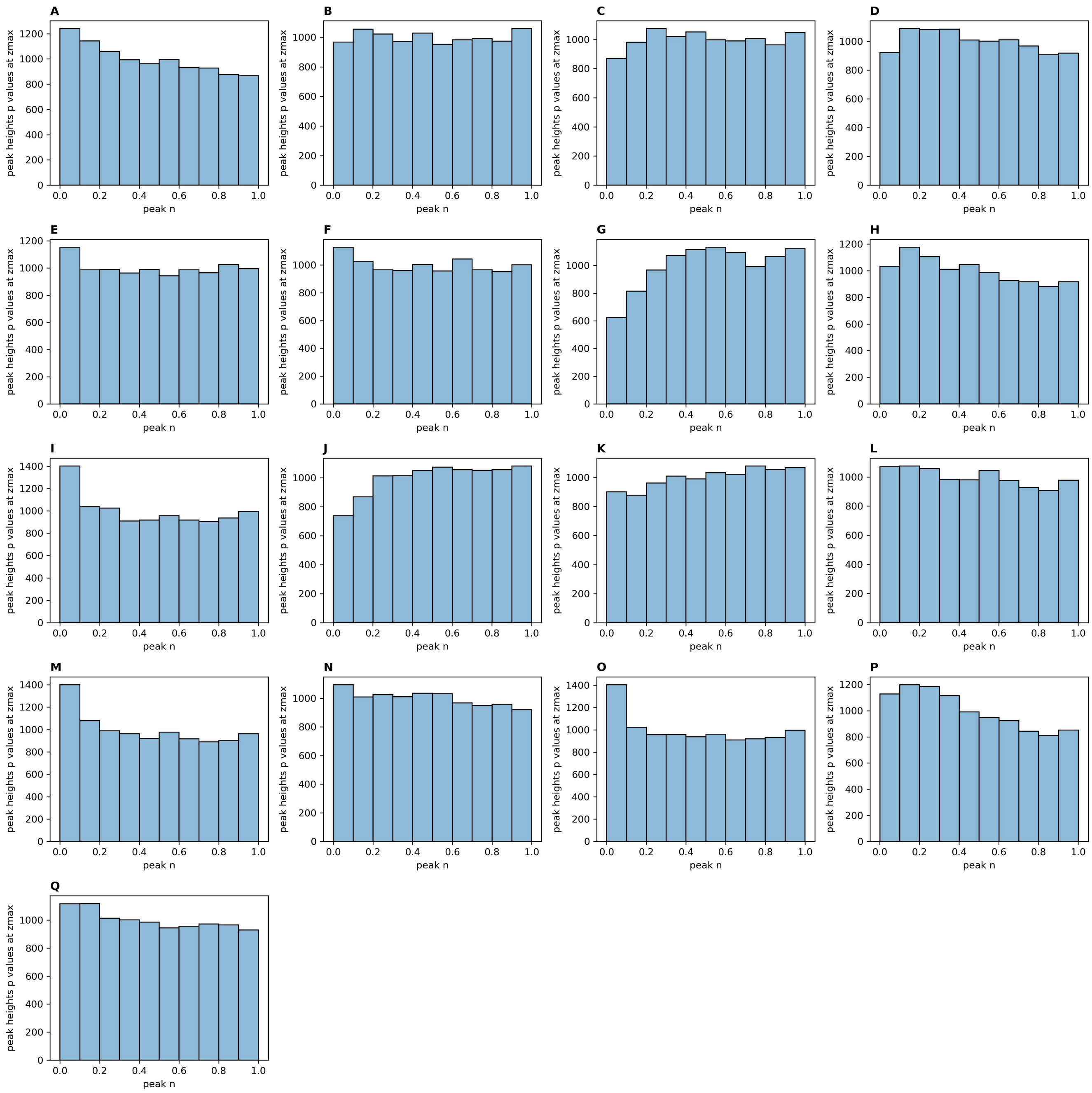

Figure S4. p value distributions of peak heights at z_{max} using the CVM goodness of fit test. **(A)** presents the results for peak 1, **(B)** for peak 2 and so on.

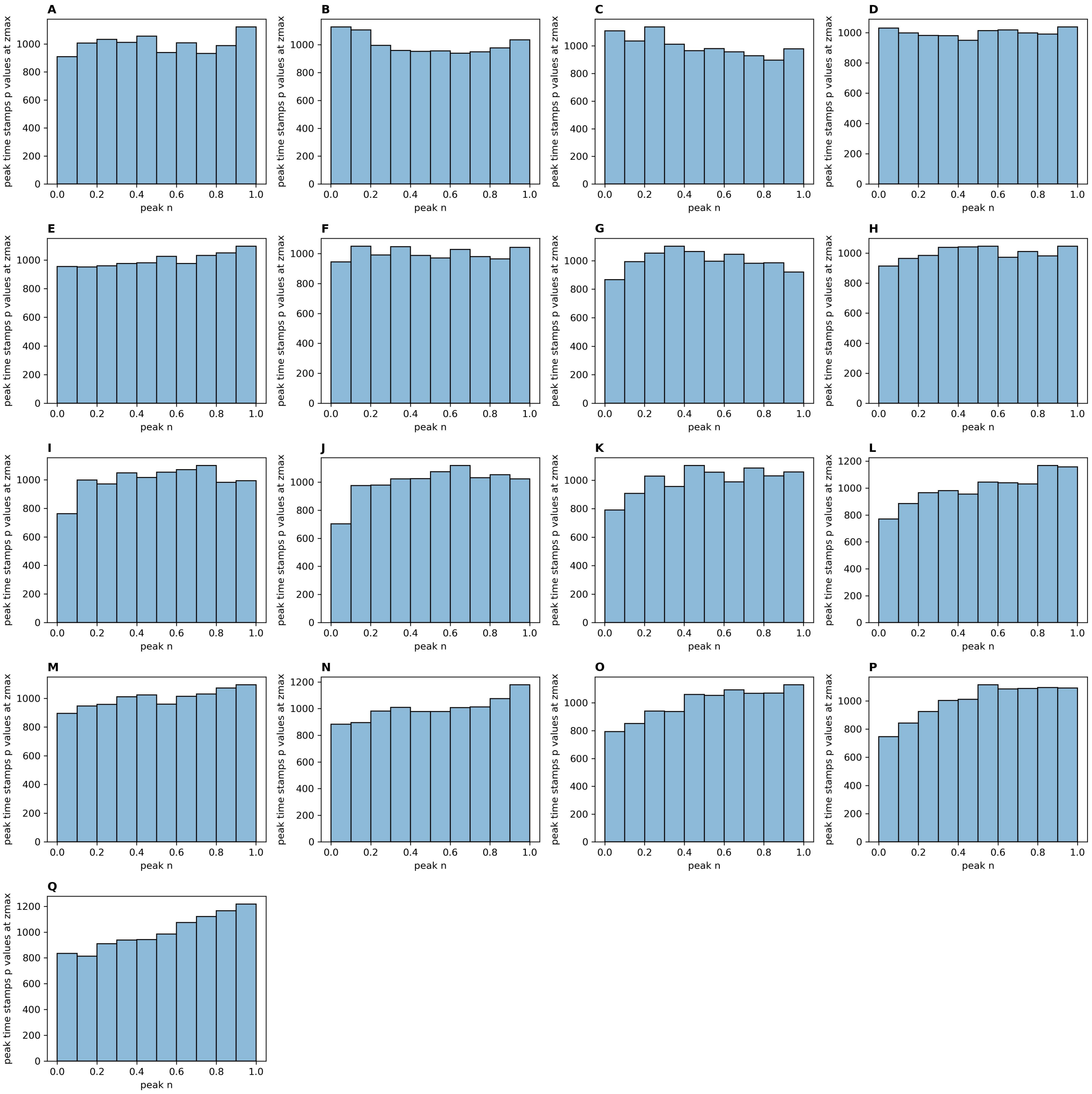

Figure S5. p value distributions of peak time stamps at z_{max} using the CVM goodness of fit test. **(A)** presents the results for peak 1, **(B)** for peak 2 and so on.

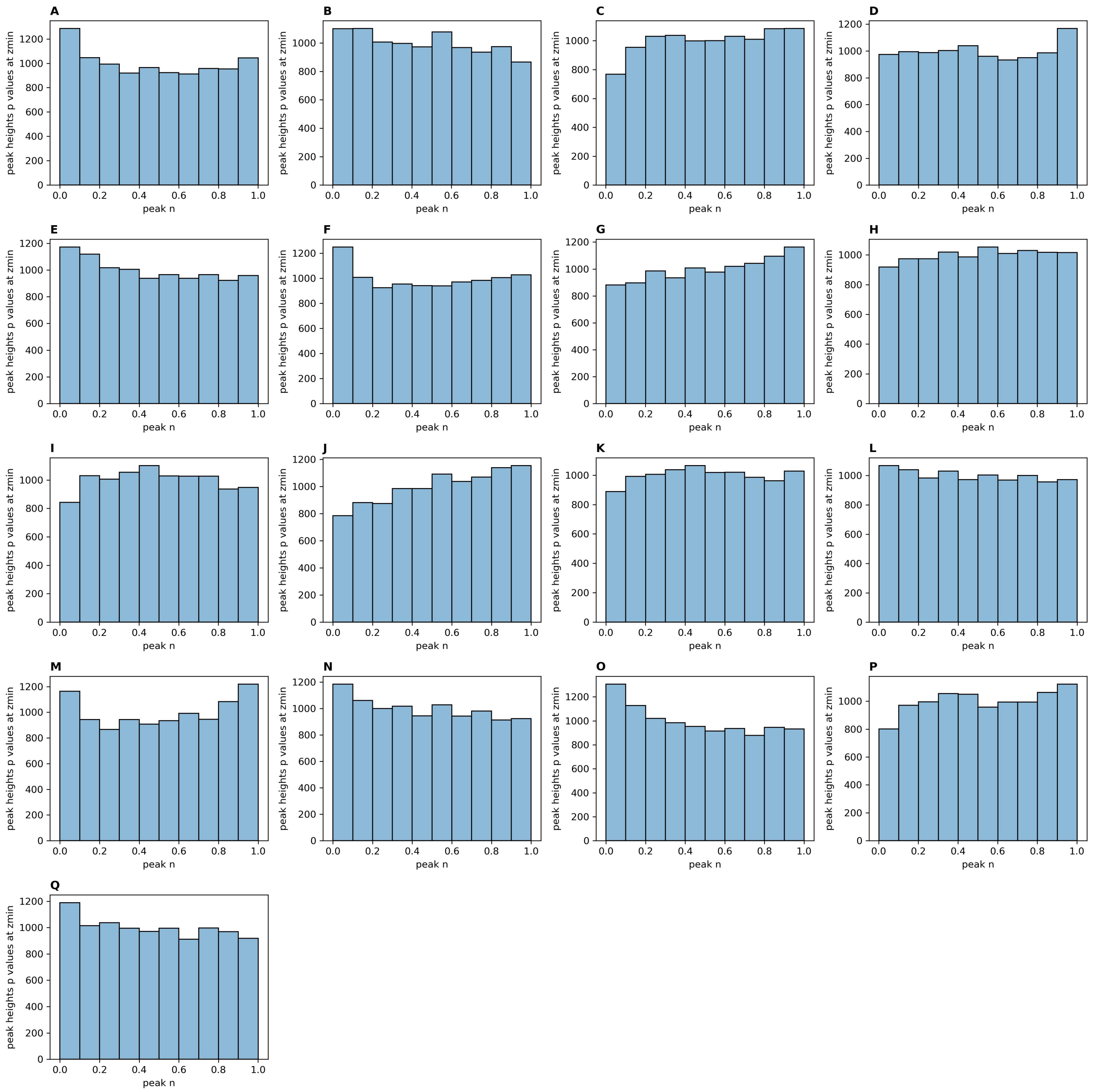

Figure S6. p value distributions of peak heights at z_{min} using the CVM goodness of fit test. **(A)** presents the results for peak 1, **(B)** for peak 2 and so on.

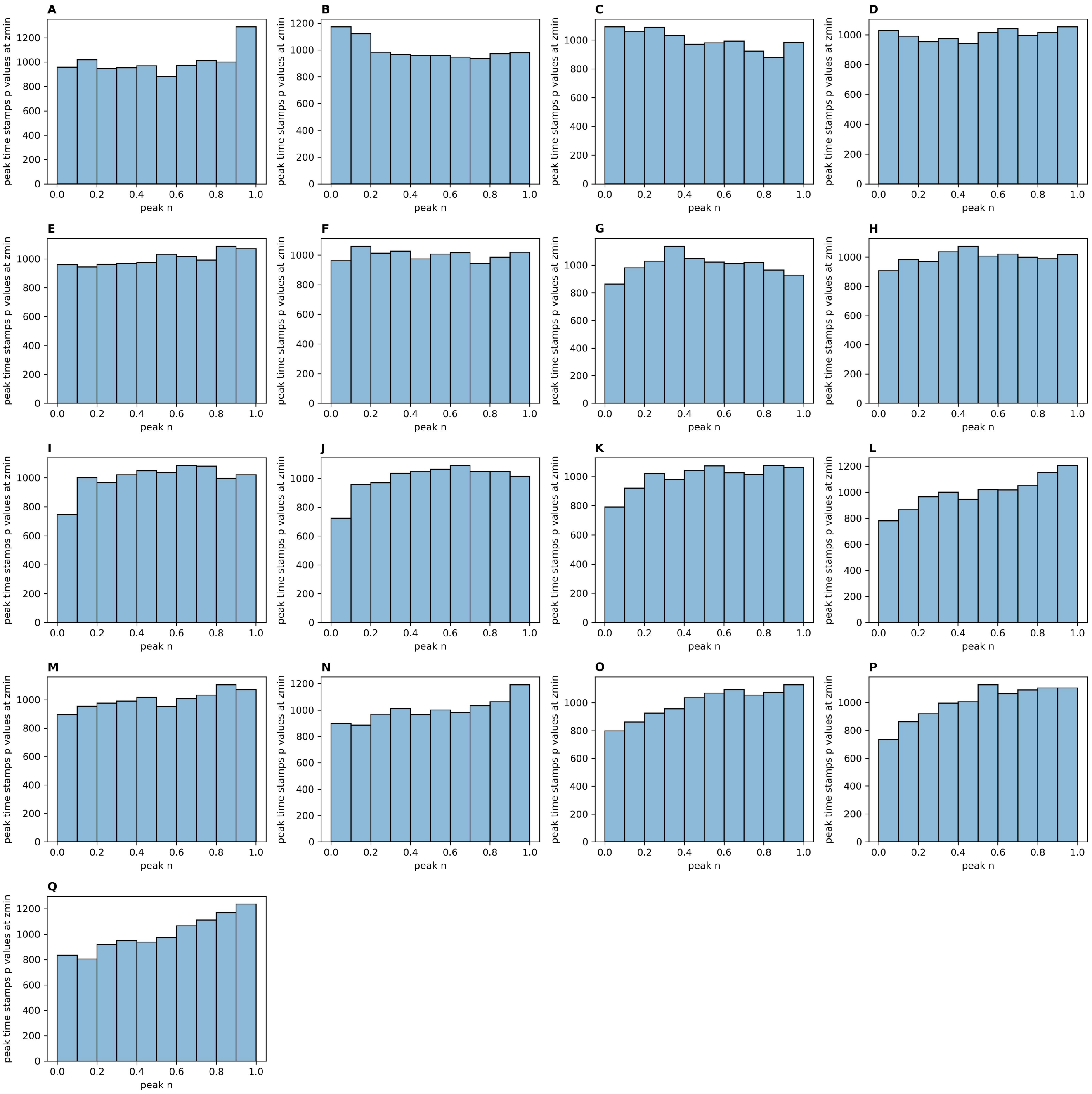

Figure S7. p value distributions of peak time stamps at z_{min} using the CVM goodness of fit test. **(A)** presents the results for peak 1, **(B)** for peak 2 and so on.

2.3 Performance

We refined the Purkinje cell mesh and reran both the calcium burst background model and the complete model. The refined mesh consists of 3,176,768 tetrahedrons and it is $\approx 3\times$ larger than the original one. Figure S8 presents the profiling of the calcium burst background model, and Figure S9 the corresponding measurements for the calcium burst complete model.

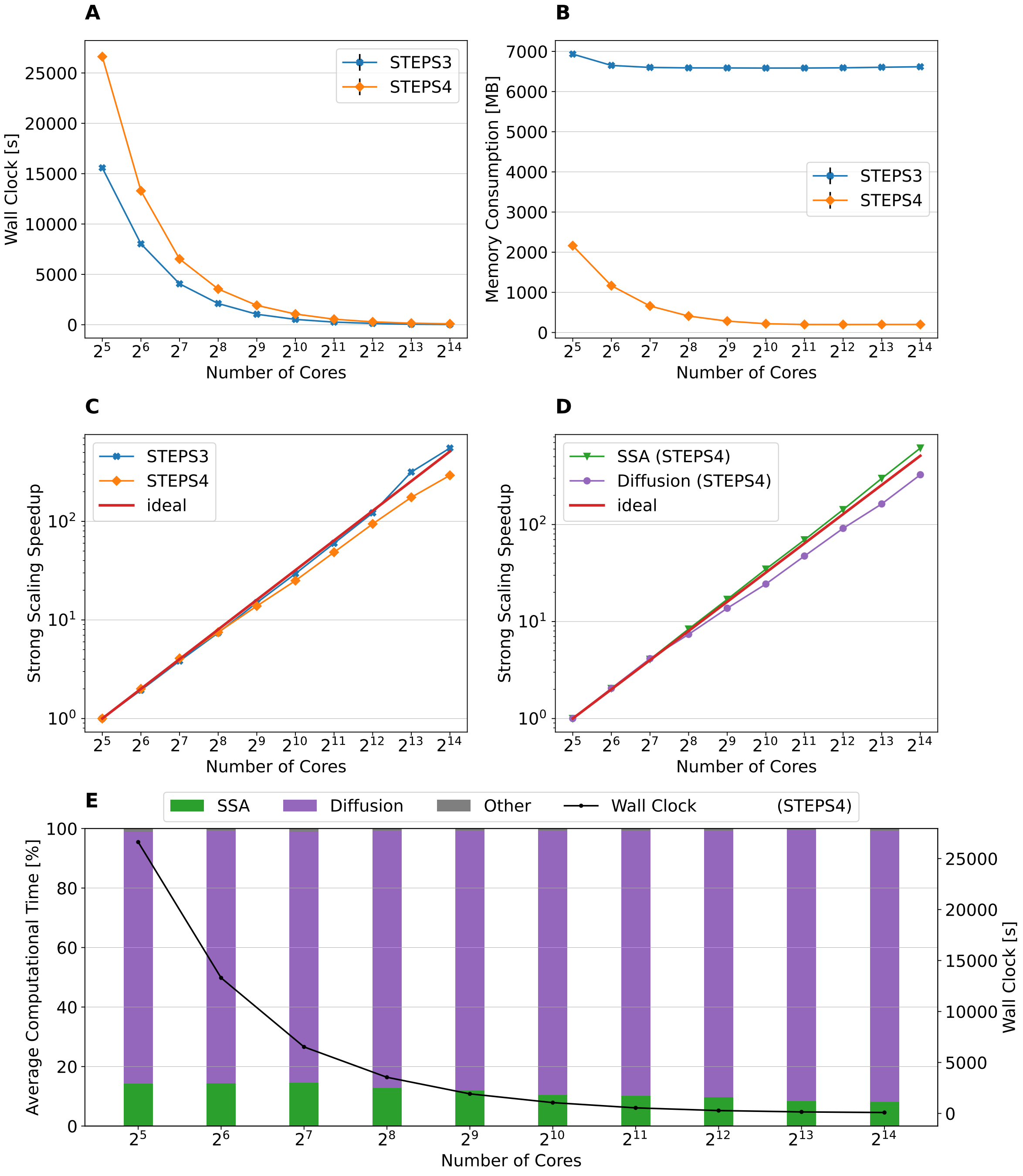

Figure S8. The performance results and scalability of the calcium burst background model for the refined mesh (denoted as "3M"). **(A)** Steady decrease of simulation time cost can be observed in both STEPS 4 and STEPS 3 simulations. STEPS 4 performs slightly worse than STEPS 3 in low core count simulations, but eventually achieves similar performance as core count increases. **(B)** The memory footprint of STEPS 4 is superior compared to the STEPS 3 counterparts, requiring about 2GB for 2^5 core simulations, and 200MB for 2^{10} core and above. STEPS 3 consumes more than 6.5GB of memory per core for the whole series. **(C)** Both STEPS 4 and STEPS 3 demonstrate linear to super-linear scaling speedup. **(D)** The diffusion operator in STEPS 4 exhibits close to linear speedup until 2^9 cores, while the SSA operator shows a remarkable super-linear speedup throughout the series. **(E)** The diffusion operator is the dominating component, taking from 85% to 90% of the overall computational time.

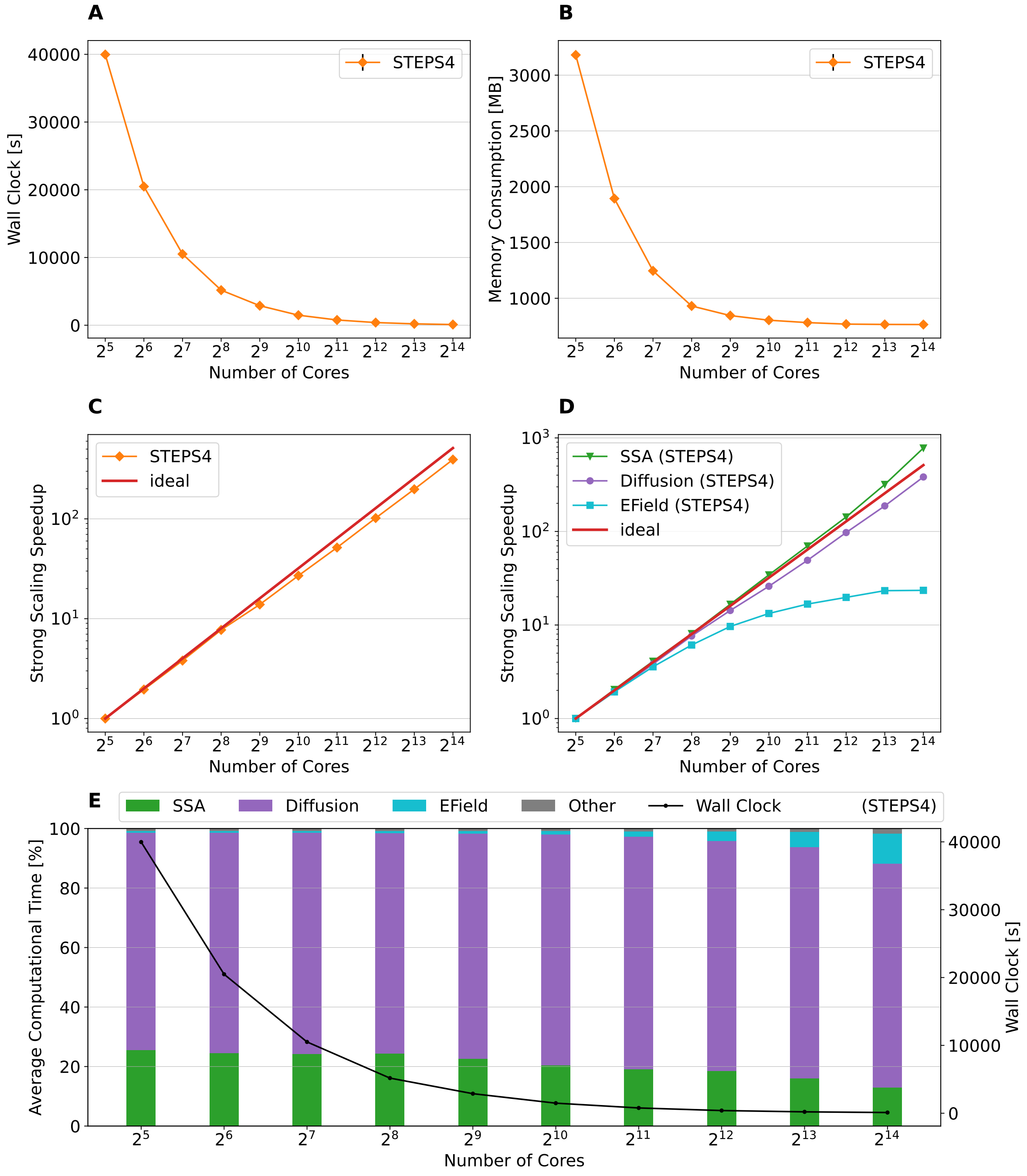

Figure S9. The performance results and scalability of the calcium burst complete model for the refined mesh (denoted as "3M"). We are unable to simulate the "3M" complete model with STEPS 3 as the 12GB per core memory quota is surpassed. **(A)** STEPS 4 Wall Clock time. **(B)** STEPS 4 memory consumption per core which is way below the 12GB per core memory quota that STEPS 3 exhausted. **(C)** STEPS 4 achieves a close to linear speedup. **(D)** The SSA operator shows a super-linear speedup throughout the series. The diffusion operator also exhibits close to linear speedup. However, the EField operator shows limited scalability. **(E)** Average Computational Time per component.

REFERENCES

Geuzaine, C. and Remacle, J.-F. (2009). Gmsh: A 3-D finite element mesh generator with built-in pre- and post-processing facilities. *International Journal for Numerical Methods in Engineering* 79, 1309–1331. doi:10.1002/nme.2579
